# Dr. Archibald Waite Bellamy

**Published:** 1913-04

**Authors:** 


Dr. Archibald Waite Bellamy died March 14, 1913, at the age of 44 years. He resided on the Ridge road in the Town of Irondequoit, and also maintained an office in the city of Rochester. He was born in Greenville County, Ontario, Canada, the son of John Bellamy; educated in the High School at Collingwood, Ontario; graduated from Queen's College, 1897, and immediately engaged in practice at Irondequoit. He remained unmarried and leaves a mother and brothers and sisters. He was a member of the A. M. A. and of the State and County Medical Societies. He was Health Officer of the Town of Irondequoit and a member of the Grange and of various fraternal organizations.

